# A family study of symbolic learning and synaptic plasticity in autism spectrum disorder

**DOI:** 10.3389/fnhum.2022.950922

**Published:** 2022-11-24

**Authors:** Guro Granerud, Torbjørn Elvsåshagen, Erik Arntzen, Katalin Juhasz, Nina Merete Emilsen, Ida Elken Sønderby, Terje Nærland, Eva Albertsen Malt

**Affiliations:** ^1^Department of Adult Habilitation, Akershus University Hospital, Oslo, Norway; ^2^Department of Behavioral Science, Oslo Metropolitan University, Oslo, Norway; ^3^KG Jebsen Centre for Neurodevelopmental Disorders, University of Oslo, Oslo, Norway; ^4^Norwegian Centre for Mental Disorders Research, Oslo University Hospital, Oslo, Norway; ^5^Department of Neurology, Oslo University Hospital, Oslo, Norway; ^6^Institute of Clinical Medicine, University of Oslo, Oslo, Norway; ^7^Department of Medical Genetics, Oslo University Hospital, Oslo, Norway; ^8^NevSom Department of Rare Disorders and Disabilities, Oslo University Hospital, Oslo, Norway; ^9^Institute of Clinical Medicine, Campus Ahus, University of Oslo, Oslo, Norway

**Keywords:** autism, 3q29, stimulus equivalence, neuropsychology, MRI, EEG

## Abstract

The current study presents a male with autism spectrum disorder (ASD) and a 3q29 deletion, and three healthy first-degree relatives. Our magnetic resonance imaging (MRI) dataset included a healthy control subset. We describe a comprehensive multimodal approach, including equivalence class formation, neurocognitive testing, MRI, and electroencephalography (EEG)-based cortical plasticity, which can provide new insights into socio-communicative and learning impairments and neural underpinnings in ASD. On neurocognitive testing, the proband showed reduced processing speed, attending behavior, and executive function. He required more training trials in equivalence class training compared with family members and exhibited impaired priming of words compared with priming with images. The proband had smaller intracranial volume and surface area and a larger visual evoked potential (VEP) C1 amplitude than family members and intact long-term potentiation (LTP)-like visual cortex plasticity. Together, these results suggest that 3q29 deletion-related ASD is associated with impaired problem-solving strategies in complex socio-communicative and learning tasks, smaller intracranial and surface area, altered VEP amplitude, and normal LTP-like visual cortex plasticity. Further studies are needed to clarify whether this multimodal approach can be used to identify ASD subgroups with distinct neurobiological alterations and to uncover mechanisms underlying socio-communicative and learning impairments.

**Lay Summary:** We studied learning, brain activity, and brain structure in a person with autism and a genetic aberration, and his close relatives. Compared with relatives, the person with autism required more training for learning, and visual learning was better than verbal learning. This person had some changes in the activity of the visual cortex, and the size and the surface area of the brain were reduced. Knowledge about learning and brain mechanisms is valuable for the development of training programs for individuals with autism.

## Introduction

Autism spectrum disorder (ASD) is a neurodevelopmental disorder characterized by difficulty with social communication and interaction, restricted interests, and repetitive behaviors (Rapin and Katzman, 1998). ASD is generally regarded as a complex genetic disorder with multifactorial causes, but several rare genetic variants conferring high risk for ASD have been identified. These genetic variants, which include copy-number variations (CNVs) arising from deletion or duplication of a genome segment, are estimated to explain about 5%–10% of the genetic risk for ASD (Ramaswami and Geschwind, 2018). Such high-risk variants present a promising opening to studying mechanisms shaping human development, cognition, and behavior (Sønderby et al., 2021).

One CNV associated with a predisposition to ASD and other neurodevelopmental disorders is the subtelomeric microdeletion of the long arm of chromosome 3, leading to 3q29 deletion syndrome (Willatt et al., 2005; Quintero-Rivera et al., 2010; Città et al., 2013; Sagar et al., 2013; Cox and Butler, 2015; Glassford et al., 2016). Haploinsufficiency of critical dose-dependent genes in the deleted region is a likely causative mechanism for ASD and various other neurodevelopmental manifestations, including facial dysmorphism, hematological alterations, and musculoskeletal abnormalities. The 3q29 deletion is of special interest because several of the 21 protein-coding genes in the deleted region are thought to have important roles in synaptic transmission and plasticity (Carroll et al., 2011; Liu et al., 2016).

Socio-communicative difficulties remain a core diagnostic feature of ASD. Effective communication requires knowledge of how symbols work, specifically that symbols refer to objects and events and can be used flexibly to represent them (Sidman, 1994). A crucial element for the successful handling of abstract concepts and symbols is the ability to understand the relations that may exist between them, such as category inclusion (A is a member of B) and relative magnitude (A is greater than B). Although people can explicitly learn or be trained on many such relations, other novel relations between concepts have been observed to emerge without training and instead as a result of exposure to an initial set of relations. For example, if the relations A–B and B–C are trained, untrained relationships between B–A, C–B, A–C, and C–A are reliably seen to emerge (Sidman, 1994; Arntzen, 2012). Sidman (1971) was the first to formalize the study of stimulus equivalence, defining the different types of derived relations as reflexivity (e.g., if A–A, then B–B), symmetry (e.g., if A–B, then B–A), and transitivity/equivalence (e.g., if A–B and B–C, then A–C and C–A).

Relations between stimuli can be directly trained in a Matching-to-Sample (MTS) procedure, followed by a test for emerged relations. In MTS, a sample stimulus and two or more comparison stimuli are presented on the computer, and participants are asked to match the correct comparison to the sample. In this way, relations are formed between stimuli that are not directly trained (Arntzen, 2012). The method is frequently used in experimental behavior analyses research, and the MTS arrangement has been successfully used to form new relations in individuals with ASD (Maguire et al., 1994; LeBlanc et al., 2003; Arntzen et al., 2010; Dixon et al., 2016).

A complementary approach to examining the formation of a relationship between stimuli is the priming procedure, which measures semantic relations or the relational strength between stimuli (Neely, 1991; Ortu et al., 2013). In this approach, related and unrelated stimulus pairs are presented. Neely (1991) demonstrated a decreased reaction time when related stimulus pairs were presented compared with presentation of unrelated pairs. This phenomenon is called the “semantic priming effect.”

When comparing participants with and without ASD, Toichi and Kamio (2001) found that the percentage of correct responses was higher for related than unrelated stimulus pairs in both groups. In support of this notion, electroencephalography (EEG) has shown different patterns of neural responses dependent on whether the participants were presented related or unrelated stimuli pairs (Bortoloti et al., 2014; Granerud-Dunvoll et al., 2019). Earlier studies were not in agreement regarding the results of semantic priming ability in participants with ASD, with some reports describing impaired ability (Minshew and Goldstein, 1993; Kamio et al., 2007) and others showing unimpaired or partially impaired ability (Bryson, 1983; Boucher, 1988; Toichi and Kamio, 2001). For instance, Kamio and Toichi (2000) found that when comparing word–word priming with picture–word priming, participants with ASD experienced a semantic priming effect in both modalities but performed better in the picture–word modality. This finding suggests that in semantic priming, pictures may offer an advantage over words for people with ASD. To our knowledge, no previous study has assessed how people with ASD respond to a priming task after MTS. Such studies can help uncover the mechanisms underlying how people with ASD engage in symbolic learning and generalization, with investigations into both the ability to form relations and the emergence of new relations and testing for association or relational strength between the trained stimuli.

The precise neural mechanisms of equivalence learning described above remains largely unknown. However, synaptic plasticity is increasingly recognized as a fundamental property of the human cortex and is thought to play central roles in learning, remembering, cognitive functioning, and emotion regulation (Pascual-Leone et al., 2005; Goto et al., 2010; Bliss et al., 2014). Thus, it can be hypothesized that synaptic plasticity processes contribute to the learning and remembering that occur during equivalence class assessments.

Results from several animal models and genetic research imply that synaptic plasticity is also involved in the development of ASD (Smoller, 2013; Martin and Manzoni, 2014; Bourgeron, 2015; Damaj et al., 2015; Li et al., 2015; Chanda et al., 2016; Jaramillo et al., 2016). Clinical studies are hampered by a lack of methods for noninvasive assessment of synaptic function, e.g., long-term potentiation (LTP), which is the best characterized form of synaptic plasticity (Malenka and Bear, 2004). However, recent developments in EEG-based experiments have opened the way to noninvasively examine plasticity that is closely related to LTP in the human visual cortex (Clapp et al., 2012; Cooke and Bear, 2012; Elvsåshagen et al., 2012). Previous investigations have shown impaired LTP-like visual cortex plasticity in affective and psychotic disorders (Normann et al., 2007; Çavuş et al., 2012; Elvsåshagen et al., 2012; Zak et al., 2018; Valstad et al., 2020), as well as in ASD (Wilson et al., 2017). The EEG-based visual plasticity experiment of the latter works was used as a window into LTP-like mechanisms in the cortex and one previous study found a significant relationship between the visual plasticity and motor cortex plasticity (Klöppel et al., 2015). Moreover, some (Bao et al., 2010), but not all (Lengali et al., 2021) studies reported significant associations between the visual plasticity and perceptual learning. Thus, to what extent EEG-based visual plasticity is linked to LTP-like processes in other cortices and learning, memory, and equivalence testing in ASD remains to be clarified.

Other neuroimaging studies suggest that ASD is also associated with alterations in brain structure, as assessed with MRI (Ecker and Murphy, 2014; Ecker et al., 2015). At the group level, children with ASD tend to have thicker frontal cortex, thinner temporal cortex, and smaller subcortical nuclei volumes when compared to typically developing children (van Rooij et al., 2018). However, findings may differ in subgroups of people with ASD (Cox and Butler, 2015).

Neuropsychology and experimental behavior analyses rely on different approaches for studying socio-communicative difficulties and symbolic processing. It is therefore of great interest to explore how the two traditions can complement each other. Neuropsychological assessments of persons with ASD also are often performed in the clinic to gain information about strengths and limitations in learning abilities and other brain functions that influence daily life. Some studies have shown that adults with ASD experience large impairments in theory of mind and emotion perception and processing and medium impairments in processing speed and verbal learning and remembering (Velikonja et al., 2019). Executive dysfunction with limitations in measures for working memory, problem-solving, flexibility, and self-control is a stable trait in people with ASD (Demetriou et al., 2018). The least altered cognitive domains seem to be attention and vigilance.

Family studies are fundamental tools in the discipline of behavioral genetics, as they permit assessments of degrees of familial resemblance, or aggregation of physical, psychological, and behavioral characteristics. Adoption studies and comparisons between monozygotic and dizygotic twins are the gold standard of these studies. In studies of rare behavioral and pleiotropic phenotypes, a supplementary approach could be to study genetic variants and phenotypes in a context that minimizes as much as possible the impact of general genetic background and environmental influences. This minimization can be achieved by studying CNV carriers together with their first-degree relatives. Such an approach could allow the generation of well-founded hypotheses about genotype-phenotype associations, which could later be tested in larger patient samples.

Here, we have conducted a study of a person with ASD and 3q29 deletion syndrome and his unaffected first-degree relatives. We applied a multimodal approach that included the stimulus equivalence paradigm, EEG-based assessment of LTP-like visual cortex plasticity, structural MRI including a larger healthy control subset, and neurocognitive testing. The overall aim of the study was to assess whether the multimodal approach can provide new insights into socio-communicative and learning impairments and their neurobiological underpinnings in ASD.

## Materials and Methods

### Participants and assessments

For this study, we included a 23-year-old male with autistic disorder and a *de novo* 1.6 Mb paternal 3q29 deletion [Arr(hg19)3q29(195747856-197339329)x1; proband], and two unaffected healthy first-degree relatives (mother and sister). The *de novo* status of the deletion was confirmed by genetic testing of the patient and the relatives. Genomic DNA from patients and first-degree relatives was extracted from peripheral blood using the DNeasy Blood and Tissue Kit (Qiagen, Hilden, Germany). DNA was sheared using a focused-ultrasonicator (Covaris, Woburn, MA, USA) and the fragment size of the sheared DNA was assessed by TapeStation (Agilent Technologies, Santa Clara, CA, USA). An Agilent SureSelect Target Enrichment System was used to capture the genomic 3q29 region, including intergenic and intragenic regions, excluding repetitive sequences, prior to sample sequencing. The 3q29 region was sequenced by MiSeq, Illumina. One brother did not complete the equivalence class formation tests but is included in the MRI part of this report. The father was not available for participation.

### Diagnostic assessments

Clinical information was collected from patients and first-degree relatives by experienced staff, including a psychiatrist, a neuropsychologist, and clinical geneticists. In the proband, ICD-10 diagnoses were assessed by a semi-structured neuropsychiatric interview, the M.I.N.I Plus International Neuropsychiatric Interview (Norwegian Translation Version 6.0.0; Lord et al., 1994), and a clinical interview with a parent. He was diagnosed with autistic disorder (ICD-10 diagnosis F84.0) at age six with the Autism Diagnostic Interview (ADI) and the Autism Diagnostic Observation Schedule (ADOS; Lord et al., 1989). The ADI is a semi-structured comprehensive parent/caregiver interview designed to evaluate early developmental history and current and lifetime presentation of autism symptomatology. The ADOS is a diagnostic, semi-structured clinical assessment that directly observes for behaviors associated with ASD in the areas of social communication, play and interaction, and restrictive and repetitive behaviors. The proband had light facial dysmorphism and musculoskeletal abnormalities (pectus excavatum, clinodactyly, kyphosis, and gait abnormality). He was underweight and suffered from chronic back pain.

The full assessment of the proband’s phenotype is described previously (see Malt et al., 2019). The first-degree relatives underwent a clinical interview that was conducted by one of the authors, who is a senior psychiatrist. The clinical interview indicated no psychiatric, neurodevelopmental, hematological, endocrine, or other severe somatic disorders. M.I.N.I. plus, ADI or ADOS were not performed in the relatives.

### Neuropsychological assessments

Verbal and nonverbal cognitive abilities were assessed with the Wechsler Adult Intelligence Scale—4th edition (WAIS-IV), the Norwegian edition with Scandinavian norms (Wechsler, 2008; Drozdick et al., 2018). Additional cognitive domains assessed included visual and verbal memory (Knox’s cube imitation test, WAIS-IV digit span, California verbal learning test-II, Rey complex figure test), processing speed (Halstead-Reitan, Trail making test A, WAIS-IV coding and symbol search), attention (Conners continuous performance test-II), executive functions (Halstead-Reitan Trail making test B, Delis-Kaplan executive function system, color-word interference test), and sensorimotor coordination (Halstead-Reitan; Grooved Pegboard).

Adaptive behavior was assessed by the semi-structured interview Vineland Adaptive Behavior Scales, 2nd edition (Sparrow et al., 1984). The relatives were not formally tested. A clinical interview including educational and occupational performances indicated normal cognitive abilities and executive function.

### Equivalence class formation

Training of conditional discriminations and testing of equivalence classes were employed and tested for as described previously (see Granerud-Dunvoll et al., 2019). In this paradigm, the proband and two family members participated. The last family member, the brother, did not respond in accordance with the predefined criterion of 90% correct responses during the testing of priming with images and was therefore excluded after the first training. This mastery criterion was set to ensure that the relations have emerged during the testing.

**Figure 1:**
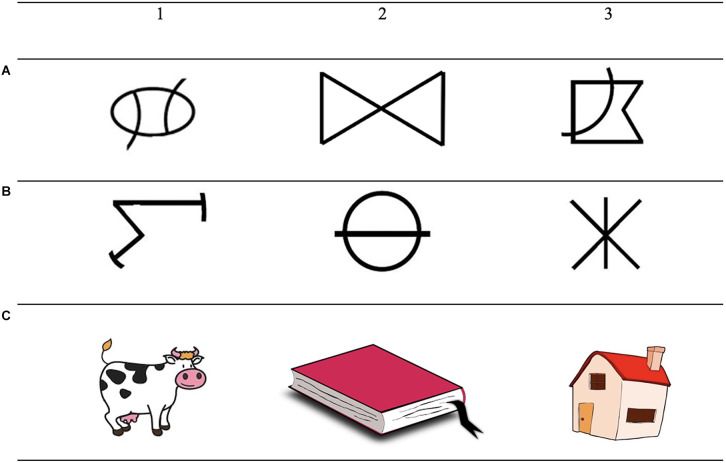
The figures show the stimuli that were used in the equivalence experiments. Note: The letters **(A–C)** vertically on the left side indicate the members of the class and the numbers (1–3) above the images indicate to which class the stimuli belong.

Both training and testing were conducted in an MTS format. Participants were trained on six conditional discriminations and tested for the emergence of three 3-member classes consisting of two abstract shapes and one familiar stimulus (Figure 1). During training, a sample stimulus was presented on the screen. The participant responded by clicking with the computer mouse on the sample stimulus. Three comparisons stimuli then appeared in the corners while the sample stimulus was still present on the screen, making this a simultaneous MTS. The participant then selected one of the comparisons stimuli, and a programmed consequence was presented on the screen, i.e., “correct” or “incorrect.” In this way, the A stimuli were trained to the C stimuli and then the B stimuli were trained to the C stimuli (15 trials per block with a 95% mastery criterion). A mix of the AC and BC relations was then presented before thinning of the programmed consequences (50%, 25%, and 0% per block each consisting of 30 trials with a 95% mastery criterion). During the test, the CA and the CB relations (symmetry) and the AB and the BA relations (transitivity/equivalence) were tested. One-third of the possible emergent relations (10 of baseline probes, 10 of symmetry probes, and 10 of equivalence/transitivity probes) was tested in the MTS format and the rest in a priming procedure. Related (20 symmetry pairs and 20 transitivity/equivalence pairs) and unrelated stimulus pairs (40 unrelated pairs) were tested by presenting pairs of stimuli successively and asking the participant to judge if the stimuli were related or not by responding on the keyboard while a question mark was present on the screen. Before the test, a training phase with the already tested symmetry and equivalence relations was conducted for rapid responding, training the participant to respond within the 2,000 ms that the question mark was presented. A word priming task was also conducted in which related and unrelated word pairs (40 of each relation) were presented. At the end, a full test for equivalence class formation was conducted (30 baseline probes, 30 symmetry probes, and 30 transitivity/equivalence probes). The criterion for correct responding in the test was set to 90% of each relation.

### Neuroimaging

**Figure 2:**
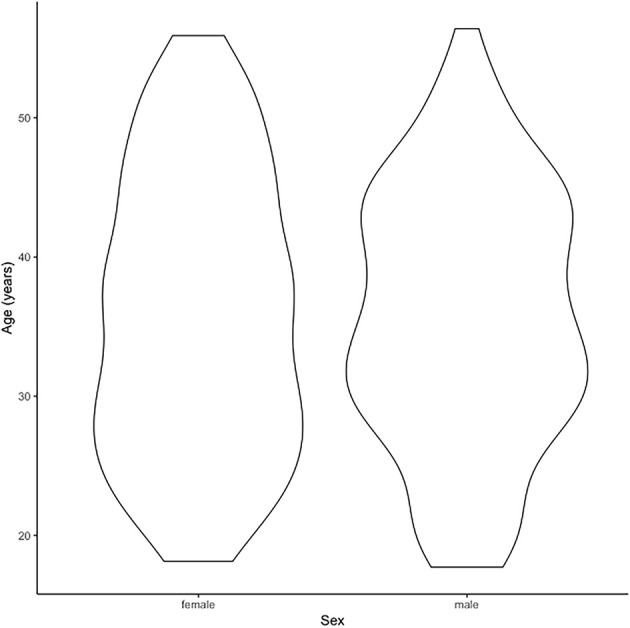
The figure indicates the age distribution of the magnetic resonance imaging (MRI) cohort of *n* = 270 healthy controls.

Our MRI dataset included a healthy control subset (*n* = 270) of the TOP study sample as described previously (Skåtun et al., 2016). In brief, structural brain MRI was performed on a GE 750 Discovery 3T magnet scanner (Kaufmann et al., 2015; Skåtun et al., 2016) and we used automated procedures for brain morphometry and volumetry^1^ (Dale et al., 1999; Fischl and Dale, 2000; Fischl et al., 2002) including cortical thickness across the brain surface. The final MRI sample of this study consisted of the 3q29 deletion carrier, his three first-degree relatives, and 270 healthy controls (125 females, 145 males, ages 18–66 years; age and sex distribution plot in Figure 2).

### LTP-like visual cortex plasticity paradigm

We assessed EEG-based LTP-like visual cortex plasticity as described recently (Valstad et al., 2020). In brief, we employed a modified version of the paradigm developed by Normann et al. (2007) in which visual evoked potentials (VEPs) in the EEG were elicited by a reversing checkerboard pattern presented on a 24-inch 144-Hz AOC LCD screen with 1-ms gray-to-gray response time. The checkerboard pattern had a spatial frequency of 1 cycle/degree and covered −28° of the visual angle (Valstad et al., 2020). The paradigm includes two baseline blocks, a plasticity-inducing modulation block, and eight postmodulation blocks. In each baseline and postmodulation block, 40 checkerboard reversals were presented within 40 s (jittered interstimulus interval of 500–1,500 ms). In the modulation block, VEPs were evoked by checkerboard reversals (using a checkerboard pattern identical to that of the baseline and postmodulation blocks and with a fixed interstimulus interval of 500 ms). The postmodulation blocks were presented at 120 s, 220 s, 380 s, 480 s, ~30 min, ~32 min, ~54 min, and ~56 min after the end of the modulation block. LTP-like visual cortex plasticity was computed by subtracting baseline VEP amplitudes from the corresponding postmodulation amplitudes. The plasticity effect is largest at the first four postmodulation blocks (Valstad et al., 2020), and VEPs of these were averaged and compared with averaged baseline VEPs in the present study.

### EEG recording and analysis

Scalp EEG data were collected from the 3q29 deletion carrier and his mother and sister. The EEG was recorded with a sampling rate of 2,048 Hz using a 64-channel BioSemi ActiveTwo amplifier system. Horizontal and vertical electro-oculograms were acquired with four bipolar electrodes placed at the sub- and supraorbital regions of the left eye and at the outer canthi of each eye. Two external electrodes were placed below the right eye, and two were placed on the right clavicle and the left iliac crest. The four latter electrodes allowed for electromyography of the right m. orbicularis oculi and electrocardiography (data not used in the present study).

The EEG data were processed using MATLAB and the EEGLAB toolbox (Delorme and Makeig, 2004). The recordings were first down-sampled to 256 Hz, and noisy channels were removed using the PREP pipeline with default settings and referenced to the channel average (Bigdely-Shamlo et al., 2015). Data were then high-pass-filtered at 1 Hz and subjected to independent component analysis to isolate blink- and eye-related activity. We applied ICLabel (Pion-Tonachini et al., 2019) to detect and reject artifactual components. The EEG data were segmented into epochs starting 100 ms before and continuing 350 ms after the onset of each checkerboard reversal. Epochs with amplitudes exceeding ± 100 μV were rejected, and the data were re-referenced to the aFz electrode and baseline-corrected (-100–0 ms). The epochs were low-pass filtered at 30 Hz and averaged to block-specific VEPs. The baseline and postmodulation VEPs presented in this study were obtained using the Oz electrode at the occipital head. We averaged the VEPs of the unaffected relatives and compared them to the baseline and postmodulation VEPs of the 3q29 deletion carrier.

## Results

### Diagnosis and neuropsychological profile

The proband had no psychiatric comorbidity. His cognitive abilities were within the lower normal range (−1.5 standard deviations: SDs). The ability profile showed significantly better results verbally (0 SD) compared with perceptually (−1.5 SD). Neuropsychological assessment examination of working, visual, and verbal memory revealed results within the normal range, whereas processing speed was reduced (−2 SD). Executive functions were impaired (−3 SD), with reductions in cognitive flexibility (Trail Making Test 4, part of the Delis-Kaplan Executive Function System; D-KEFS; Delis et al., 2001) and speed of mental processing (Color word 1–3; D-KEFS; Delis et al., 2001). His scores also indicated problems with attention. Adaptive behavior was significantly impaired (−2.5 SD) and indicated a significant need for care in everyday life.

### Symbolic learning: equivalence class formation

#### MTS training and test 1

**Figure 3:**
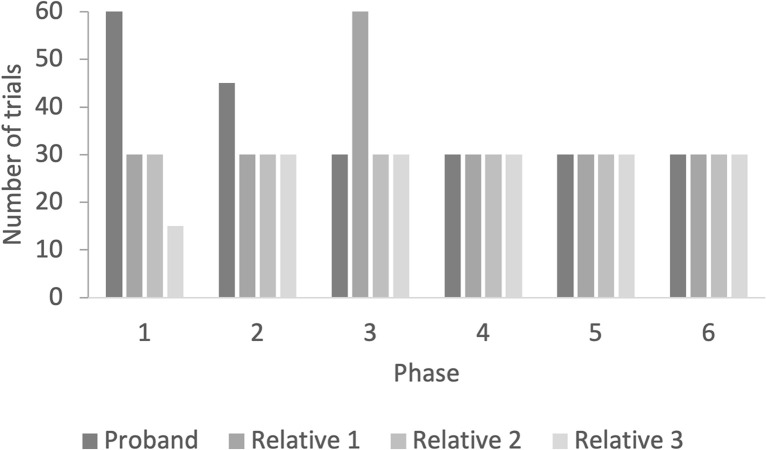
The figure shows the number of training trials in the AC-training, BC-training, AC/BC mix-training, and mix-training with 50%, 25%, and 0% fading of programmed consequences.

The proband used 225 training trials to establish the relations (Figure 3). In the first test, with one-third of the relations, he responded in accordance with equivalence (100%). The three unaffected family members had an average of 185 training trials to establish the relations. Relative 1 used 210 training trials, relative 2 used 180 training trials, and relative 3 used 165 training trials (Figure 3). All relatives responded in accordance with equivalence in the one-third equivalence test (100%).

#### Priming images

**Figure 4:**
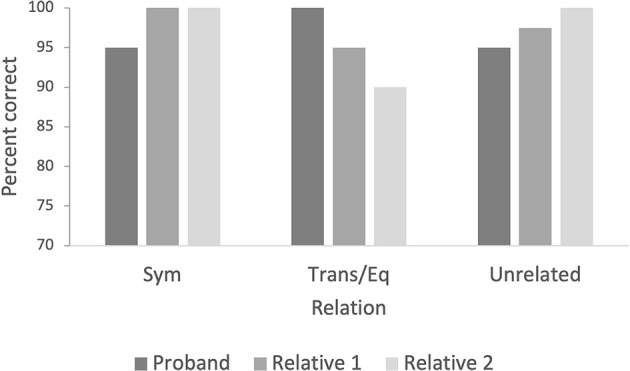
The figure indicates the percentage of correct responses for symmetry, transitivity/equivalence, and unrelated pairs for the priming test with images. Note. BP, baseline probes; Sym, symmetry; Trans, transitivity; Eq, equivalence.

The proband had 95% correct symmetry relations, 100% correct transitivity/equivalence relations, and 95% correct unrelated relations (Figure 4). The unaffected family members both had 100% correct responses to the symmetry relations (100% for both relatives), average of 92.5% correct responses for transitivity/equivalence (95% for relative 1 and 90% for relative 2), and average of 98.7% correct responses for the unrelated relations (97.5% for relative 1 and 100% for relative 2; Figure 4). The last unaffected family member responded under the criterion of 90% in this phase and was excluded.

#### Priming words

**Figure 5:**
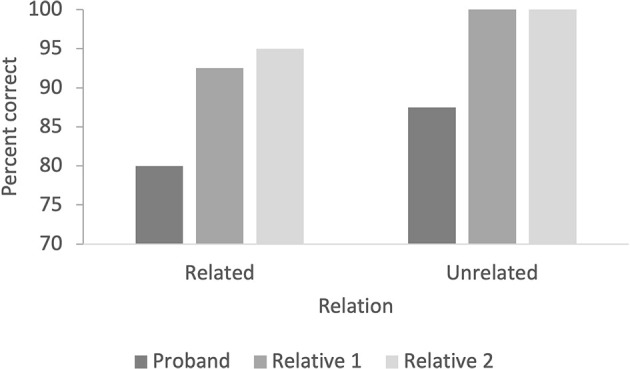
The figure indicates the percentage of correct responses in related and unrelated pairs for the priming test with words.

The proband carrier scored 80% correct for the related pairs and 87.5% for the unrelated pairs (Figure 4). The unaffected family members averaged 93.7% correct responses to the related pairs (92.5% for relative 1 and 95% for relative 2), and both had 100% correct responses for unrelated pairs (Figure 5).

#### MTS test 2

The proband responded in accordance with baseline probes (100%), symmetry (100%), and transitivity/equivalence (100%). The two unaffected family members responded in accordance with baseline probes (100% for both), symmetry (100% for both), and transitivity/equivalence (average 96.6%: 100% for relative 1 and 93.3% for relative 2).

### Neuroimaging

Using MRI, we investigated if the 3q29 carrier showed structural brain differences from his family and the 270 healthy controls. No brain abnormalities were observed in any of the unaffected family members. We investigated the within-sex percentiles relative to the healthy control population for each family member for the following global MRI measures: intracranial volume, total cortical surface area, and mean cortical thickness. These analyses showed that the 3q29 carrier had smaller intracranial volume and total cortical surface area relative to the healthy controls (25th and 14th percentiles, respectively) and the unaffected family members (Table 1).

**Table 1:** Percentiles in a magnetic resonance imaging dataset of *n* = 270 healthy controls.

	**Age**	**Intracranial volume**	**Cortical surface area**	**Cortical mean thickness**
3q29 carrier	23.2	25%	14%	36%
Brother	28.0	86%	97%	18%
Sister	20.9	77%	90%	54%
Mother	51.9	95%	95%	23%

Percentiles were calculated separately on males and females.

### LTP-like visual cortex plasticity and baseline VEP amplitudes

**Figure 6:**
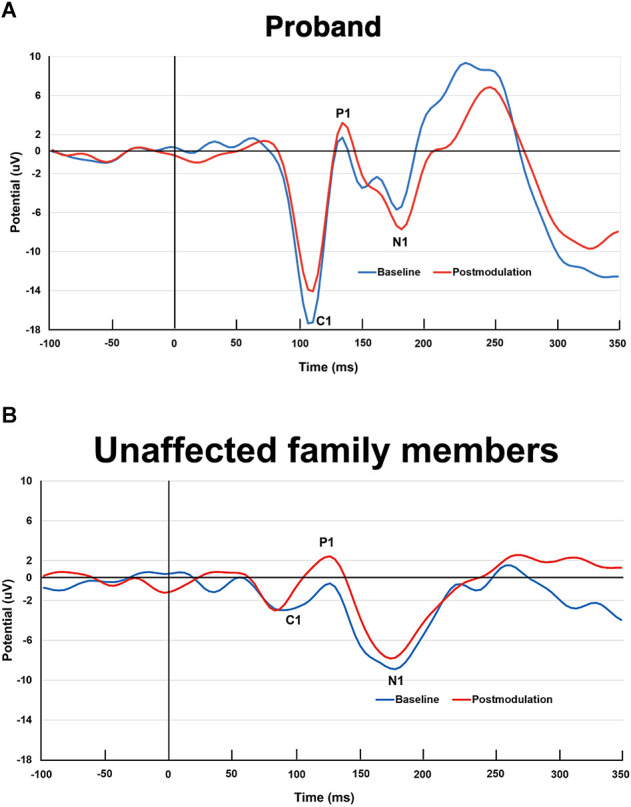
**(A)** Visual evoked potentials (VEPs) elicited by checkerboard reversal stimulation at baseline (blue color) and postmodulation (i.e., after a plasticity inducing intervention of prolonged visual stimulation for 10 min; red color) in the 3q29 deletion carrier. Note. This paradigm induces long-term potentiation (LTP)-like plasticity in the early visual cortices, as recently demonstrated in a larger sample of healthy volunteers (Valstad et al., 2020; NeuroImage). The 3q29 deletion carrier of the present study exhibited the expected pre-post change, i.e., decreased C1 amplitude and increased P1 and N1 amplitudes. **(B)** Visual evoked potentials elicited by checkerboard reversal stimulation at baseline (blue color) and postmodulation (i.e., after a plasticity inducing intervention of prolonged visual stimulation for 10 min; red color) in the unaffected family members. Note. The family members showed the expected pre-post P1 VEP amplitude increase, yet no clear changes in C1 or N1 amplitudes.

The proband and his unaffected family members showed the expected C1, P1, and N1 amplitudes of the VEP at the baseline and postmodulation blocks (Figure 6). In a comparison of postmodulation and baseline VEPs, the proband exhibited a decrease in the C1 amplitude, with P1 and N1 amplitude increases (Figure 6A), consistent with the LTP-like effects recently described in a large sample of healthy volunteers (Valstad et al., 2020). The unaffected family members had increased P1 amplitude at the postmodulation blocks, whereas the C1 and N1 changes from baseline to postmodulation were less pronounced (Figure 6B). The baseline C1 amplitude of the 3q29 deletion carrier was larger than the averaged C1 amplitude of his unaffected family members and of the group of healthy volunteers (Valstad et al., 2020).

## Discussion

This study is the first to combine methods spanning from behavior analysis to novel approaches in synaptic plasticity research to examine symbolic learning in ASD. The main result is that the proband showed impairment in the handling of abstract concepts and symbols. He needed more trials than his first-degree relatives to form equivalence classes, and the responses to word-priming were especially impaired. These results supplement neuropsychological findings of attention deficits, reduced processing speed, and reduced cognitive flexibility. The proband also had lower percentiles for intracranial volume and cortical surface area compared with his family members. LTP-like visual cortex plasticity was not impaired, but the amplitude of the C1 component of the VEP, reflecting the initial afferent activity in primary visual cortex, was larger at baseline in proband than in the relatives.

### Equivalence class formation

The results show that the proband, despite responding as accurately as his relatives on the test for equivalence class formation and the priming test with images, required more trials to learn the trained relation before the subsequent testing and did not respond according to the mastery criterion of 90% in word-priming. He did not demonstrate impaired priming results with images compared to his relatives, in keeping with findings of Toichi and Kamio (2001). However, neither the first-degree relatives nor the proband demonstrated the semantic priming effect (higher percent correct responses to related stimulus pairs compared to unrelated pairs). All three participants reached the mastery criterion of 90% with both related and unrelated stimulus pairs with images. No semantic priming effect based on percent correct was detected in the word priming paradigm, either. However, only the proband responded under the mastery criterion. These results are consistent with those of Toichi and Kamio (2001), who reported that participants with ASD performed significantly better in the picture–word priming task than in the word–word priming task. This pattern was evident in the current study, even though the participant with ASD scored within the normal field in working memory and visual and verbal memory on neurocognitive testing.

One possible reason for these findings is the short presentation time for the prime and target. In neurocognitive testing, he showed reduced processing speed, attentional problems, and reduced cognitive flexibility and speed of mental processing. The participant was diagnosed with cognitive abilities within the lower range, which could partly explain the need for more training trials in MTS, but in test of equivalence class formation, he responded at the same level as the family members where there was no suspicion of lower cognitive abilities. He also responded correctly in about 80% of the relations in word priming, which indicates that reduction in process speed, attention, and executive function might at least partly correlate with impairments in word priming. When images were presented as prime and target, the effect to respond could be lower than that for written words, which can be more complex stimuli than images, even though the images were not directly trained. Written words could also demand increased attention to stimuli compared to images. In addition, the images were known to the participant because they were presented during the MTS training and testing. Altogether, the results from the neurocognitive tests and tests of equivalence class formation support the suggestion made by Toichi and Kamio (2001) that in people with autism, thinking may be a predominantly visual rather than verbal. A potential practical consequence is that visual aids might strengthen verbal instructions in learning situations for individuals with ASD.

### Neuroimaging

The proband had smaller intracranial volume and cortical surface area compared with his family members, as shown by lower percentiles relative to a healthy control population. The small intracranial volume is consistent with previous findings of microcephaly in 3q29 deletion carriers (Cox and Butler, 2015), and our analyses are the first to suggest that this feature may correlate with reduced cortical surface area. The 3–4 SD reductions in intracranial volume and cortical surface area of our proband in comparison to a healthy population may suggest a large effect of the 3q29 deletion on these measures.

### LTP-like visual cortex plasticity

We found that the proband had LTP-like visual cortex plasticity comparable to that of healthy controls (Valstad et al., 2020). Clearly, no definitive conclusions can be drawn from results for one individual, yet this finding may suggest that the 3q29 deletion in this case, at least, did not impair LTP-like plasticity in the early visual cortices. We are not aware of previous studies of LTP-like plasticity in individuals with the 3q29 deletion, and further studies are needed to clarify whether and how this deletion affects plasticity in the visual and other cortical regions. Visual cortex plasticity studies in ASD are lacking in general, but a recent report of 12 adults with ASD and 12 controls found evidence for increased LTP-like visual plasticity in the ASD group (Wilson et al., 2017). These researchers employed an EEG-based visual cortex plasticity paradigm that was similar but not identical to the one used in the current work and detected greater postmodulation increase in the N1 amplitude in the ASD group vs. controls. However, other studies in people with ASD have detected impaired LTP-like plasticity in the motor cortices (Jung et al., 2013; Pedapati et al., 2016). Further investigations of plasticity in the visual and other cortices of individuals with ASD are clearly warranted.

We also note that the proband had a larger C1 amplitude than his unaffected family members at baseline, i.e., before induction of the LTP-like effect. Although there are no previous reports of VEP amplitudes in 3q29 deletion syndrome, several earlier works have assessed checkerboard-reversal VEPs in ASD. One such study detected reduced P1 amplitude in children with ASD, ages 42–130 months, compared with typically developing children (Kovarski et al., 2019). The authors did not find any significant group differences when adjusting for inter-trial variability, however, which was pronounced in a subset of the children with ASD. These observations suggest that apparent VEP amplitude reductions in ASD might be related to increased inter-trial variability (e.g., more trials without clear visual cortex responses) and that VEP amplitudes may vary between subgroups of ASD. Consistent with these findings, another group reported increased intra-participant visual P1 amplitude variability in children and adolescents with ASD relative to neurotypical matched controls, yet without amplitude differences between the groups (Milne, 2011). Another study of children with ASD, ages 3–5 years, found no significant differences when comparing checkerboard-reversal VEP amplitudes with those of age-matched controls (Sayorwan et al., 2018). Finally, a report concerning checkerboard-reversal VEPs in adolescents and adults with ASD described reduced P1 amplitude in patients when compared with controls (Kovarski et al., 2016). These authors did not adjust their analyses for inter-trial variability, however. Thus, further studies that do make these adjustments are needed to characterize VEP alterations in ASD and to clarify whether an increased visual C1 amplitude is a specific biomarker of 3q29 deletion syndrome and other subgroups of ASD. If these features can be confirmed in future studies, it would be interesting to explore whether the amplitude increase is associated with the hypersensitivity to sensory stimuli that is commonly reported for individuals with ASD (Takarae et al., 2016).

## Limitations

The main limitation of the present study is that only one patient with the 3q29 deletion syndrome was included. Thus, no definitive conclusions can be drawn from the results from the affected individual and larger studies are needed to test the hypotheses generated by this work.

## Conclusion

The 3q29 deletion carrier with ASD showed impairment in the handling of abstract concepts and symbols, reduced measures of cognitive flexibility and speed of mental processing in cognitive testing, reductions in intracranial volume and cortical surface area, and increased visual C1 amplitude. By taking brain structure and function and observable problem-solving behaviors into account, the findings of this broad investigation relying on neurocognitive testing and tests for equivalence class formation and priming, MRI, and EEG may help reveal mechanisms interfering with the environment in socio-communicative and learning impairments in ASD and other neurodevelopmental conditions. Improved knowledge of brain structure and function and problem-solving behavior may aid us in devising individually targeted, personalized interventions in 3q29 deletion carriers and contribute to our understanding of ASD in general. Further studies are needed to clarify whether this multimodal approach can assist in differentiating subgroups of people with autism, providing valuable information for personalized goal-directed interventions and training programs.

## Data Availability Statement

The raw data supporting the conclusions of this article will be made available by the authors, without undue reservation.

## Ethics Statement

The studies involving human participants were reviewed and approved by Regional Committees of South-Eastern Norway for Medical and Health Research Ethics (2016/629). The patients/participants provided their written informed consent to participate in this study. All participants were informed that they could withdraw at any time from the study without any negative consequences.

## Author Contributions

GG: conceptualization, methodology, investigation, formal analysis, visualization, writing—original draft, writing—review and editing. TE: conceptualization, methodology, supervision, software, formal analysis, visualization, funding acquisition, writing—original draft, writing—review and editing. EA: conceptualization, methodology, supervision, software, formal analysis, visualization, writing—original draft, writing—review and editing. KJ and NE: methodology, investigation, writing—original draft. IS: methodology, investigation, formal analysis, visualization, writing—original draft. TN: funding acquisition, writing—review and editing. EM: conceptualization, methodology, supervision, funding acquisition, writing—original draft, writing—review and editing.

## Funding

This study was funded by the South-Eastern Norway Regional Health Authority (#2015-078), Oslo University Hospital, Akershus University Hospital, Oslo Metropolitan University, the Ebbe Frøland foundation, and a research grant from Mrs. Throne-Holst. IS is supported by the Research Council of Norway (#223273), South-Eastern Norway Regional Health Authority (#2020060), the European Union’s Horizon 2020 Research and Innovation Programme (CoMorMent project; Grant #847776), and the Kristian Gerhard Jebsen Stiftelsen (SKGJ-MED-021). TE is supported by the South-Eastern Norway Regional Health Authority (#2015078, #2021041). Services for sensitive data, University of Oslo, Norway, with resources provided by UNINETT Sigma2—the National Infrastructure for High Performance Computing and Data Storage in Norway provided data storage.

## Footnotes

^1^ http://surfer.nmr.mgh.harvard.edu
